# Role of microRNAs in epigenetic silencing of the *CHD5* tumor suppressor gene in neuroblastomas

**DOI:** 10.18632/oncotarget.7434

**Published:** 2016-02-16

**Authors:** Koumudi Naraparaju, Venkatadri Kolla, Tiangang Zhuang, Mayumi Higashi, Radhika Iyer, Sriharsha Kolla, Erin R. Okawa, Gerd A. Blobel, Garrett M. Brodeur

**Affiliations:** ^1^ Division of Oncology and Hematology, The Children's Hospital of Philadelphia, University of Pennsylvania, Philadelphia, PA, USA; ^2^ Department of Pediatrics, The Children's Hospital of Philadelphia, University of Pennsylvania, Philadelphia, PA, USA

**Keywords:** neuroblastoma, CHD5, miRNA, tumor suppressor, *MYCN*

## Abstract

Neuroblastoma (NB), a tumor of the sympathetic nervous system, is the most common extracranial solid tumor of childhood. We and others have identified distinct patterns of genomic change that underlie diverse clinical behaviors, from spontaneous regression to relentless progression. We first identified *CHD5* as a tumor suppressor gene that is frequently deleted in NBs. Mutation of the remaining *CHD5* allele is rare in these tumors, yet expression is very low or absent, so expression is likely regulated by epigenetic mechanisms. In order to understand the potential role of miRNA regulation of *CHD5* protein expression in NBs, we examined all miRNAs that are predicted to target the 3′-UTR using miRanda, TargetScan and other algorithms. We identified 18 miRNAs that were predicted by 2 or more programs: miR-204, -211, -216b, -17, -19ab, -20ab, -93, -106ab, -130ab, -301ab, -454, -519d, -3666. We then performed transient transfections in two NB cell lines, NLF (*MYCN* amplified) and SY5Y (*MYCN* non-amplified), with the reporter plasmid and miRNA mimic, as well as appropriate controls. We found seven miRNAs that significantly downregulated *CHD5* expression in NB: miR-211, 17, -93, -20b, -106b, -204, and -3666. Interestingly, *MYCN* upregulates several of the candidates we identified: miR-17, -93, -106b & -20b. This suggests that miRNAs driven by *MYCN* and other genes represent a potential epigenetic mechanism to regulate *CHD5* expression.

## INTRODUCTION

Neuroblastoma (NB) is the most common extracranial solid tumor of childhood. NBs show clinical heterogeneity, from spontaneous regression to relentless progression, and NBs account for a disproportionate number of childhood cancer deaths. We and others have identified different patterns of genomic change that underlie these contrasting clinical behaviors [1–6]. Deletion of the short arm of chromosome 1 (1p) occurs in 35% of primary tumors and 80% of tumor-derived cell lines, representing one of the most characteristic genomic changes in NBs [7–10]. Presumably, 1p deletion reflects loss of a tumor suppressor gene (TSG) from this region. We mapped the smallest region of consistent deletion (SRD) to an approximately 2 Mb region on 1p36.31 [11, 12]. Indeed, the SRD identified by most other groups mapping 1p deletions in NBs overlaps our region [13–16]. We analyzed 23 genes mapping to the maximal SRD on 1p36.31, and we identified *CHD5* as the most likely TSG within this region [11, 17, 18].

The *CHD5* gene encodes a novel member of the chromodomain helicase DNA binding (CHD) family, and all proteins have nuclear localization signals, paired chromodomains as well as ATP-dependent helicases [19]. Increasing evidence suggests that CHD protein complexes play an important role in regulating development, cell cycle control and oncogenesis through their influence on chromatin structure and gene expression [20]. *CHD5* is expressed almost exclusively in the nervous system and in testis, and expression is virtually undetectable in a panel of NB cell lines compared with fetal brain [21–23].

DNA methylation of the *CHD5* promoter region has been well documented in NBs and many other tumor types [17, 24–29]. However, we are exploring other important epigenetic mechanisms of *CHD5* transcriptional/translational regulation, including microRNAs (miRNAs). MiRNAs are small, non-coding RNA molecules that have a seed region 2-7 nucleotides from the 5′ end of the miRNA. The seed region imperfectly pairs with the 3′-untranslated region (3′-UTR) of the target mRNA [30]. Binding may then result in mRNA cleavage, degradation or reduced translation efficiency at the ribosome [31]. The imperfect pairing between a miRNA and its target allows a given miRNA to bind one or more sites within a 3′-UTR, or to multiple mRNA targets throughout the genome. This allows miRNAs to have a robust influence on gene expression, and it allows simultaneous regulation of multiple components of the signaling network in development and in cancer [32].

In the present study, we investigated the role of miRNAs on downregulation of *CHD5* in NB cell lines *in vitro*. We used computational analysis to predict which miRNAs may base pair with the 3′-UTR of *CHD5* and performed a functional assay to confirm which of the miRNAs target *CHD5*. We also examined the expression of these miRNAs in primary tumors. Finally, we performed functional assays by transfecting miRNA mimics into a cell line with endogenous *CHD5* expression to determine if the miRNAs identified by our reporter assay were able to decrease *CHD5* protein expression.

## RESULTS AND DISCUSSION

### We identified 18 miRNAs that were predicted to target *CHD5*

**Figure 1:**
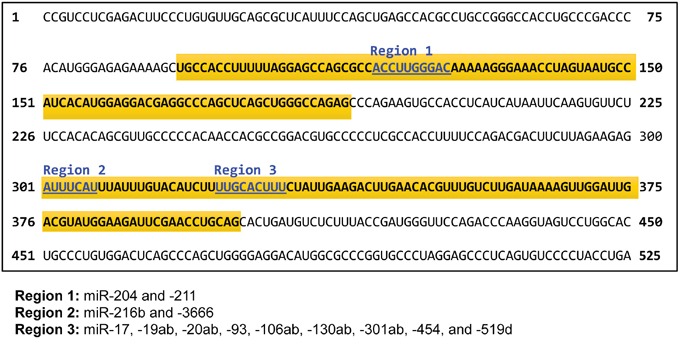
Nucleotide sequence of the 3′ untranslated region (UTR) of *CHD5* Three locations that microRNAs are predicted to bind in the *CHD5* 3′-UTR are shown in blue, and the miRNAs that target each of these regions are shown below. The regions cloned into the targeting vector are highlighted in yellow.

We used miRanda (www.microrna.org), TargetScan (www.targetscan.org), miRDB (mirdb.org), and DIANA 3.0 (diana.cslab.ece.ntua.gr/microT) to identify miRNAs predicted to target the *CHD5* 3′-UTR. Each program yielded a unique list, so we focused on the miRNAs identified by at least two of these prediction algorithms. We identified 18 miRNAs that bound to one of three different target regions of the *CHD5* 3′-UTR: Region 1 bound miR-204 and -211; Region 2 bound miR-216b and -3666; and Region 3 bound miR-17, -19ab, -20ab, -93, -106ab, -130ab, -301ab, -454, and -519d (Figure 1). The miRNAs, as well as their chromosomal location and nucleotide sequence, are shown in Supplementary Table S1. We focused on these miRNAs for further functional analysis in the NLF and SY5Y NB cell lines. Interestingly, others showed that eight of these miRNAs are *MYCN* driven in NBs, including miR-17, -19a, -19b, -20a, -20b, -93, -106a, and -106b [33–35].

### Select *MYCN*-driven and other miRNAs directly downregulate *CHD5* 3′-UTR reporter

For our transfection studies, *Renilla* luciferase signals were first normalized to an internal *firefly* luciferase transfection control. Then all samples were normalized using the Qiagen Allstars siRNA as a negative control. There was a prior report that miR-211 targeted the *CHD5* 3′-UTR in colorectal cancer [36], so we used miR211 as a positive control for these studies. Indeed, all transient transfections were screened for a significant reduction in the wild-type (WT) *CHD5* 3′-UTR compared to no *CHD5* 3′-UTR and mutant *CHD5* 3′-UTR constructs when using the miR-211 mimic. Similar values were obtained for all three vectors when transfected with no miRNA mimic (transfection control). For miRNAs that targeted *CHD5*, we expected to see a lower ratio for the WT *CHD5* 3′-UTR compared to both the no insert and mutant *CHD5* 3′-UTR controls. Data from at least three independent experiments, each done in triplicate, were analyzed using the Prism two-way ANOVA method, followed by a Sidak post-test, as described in the Materials and Methods.

**Figure 2:**
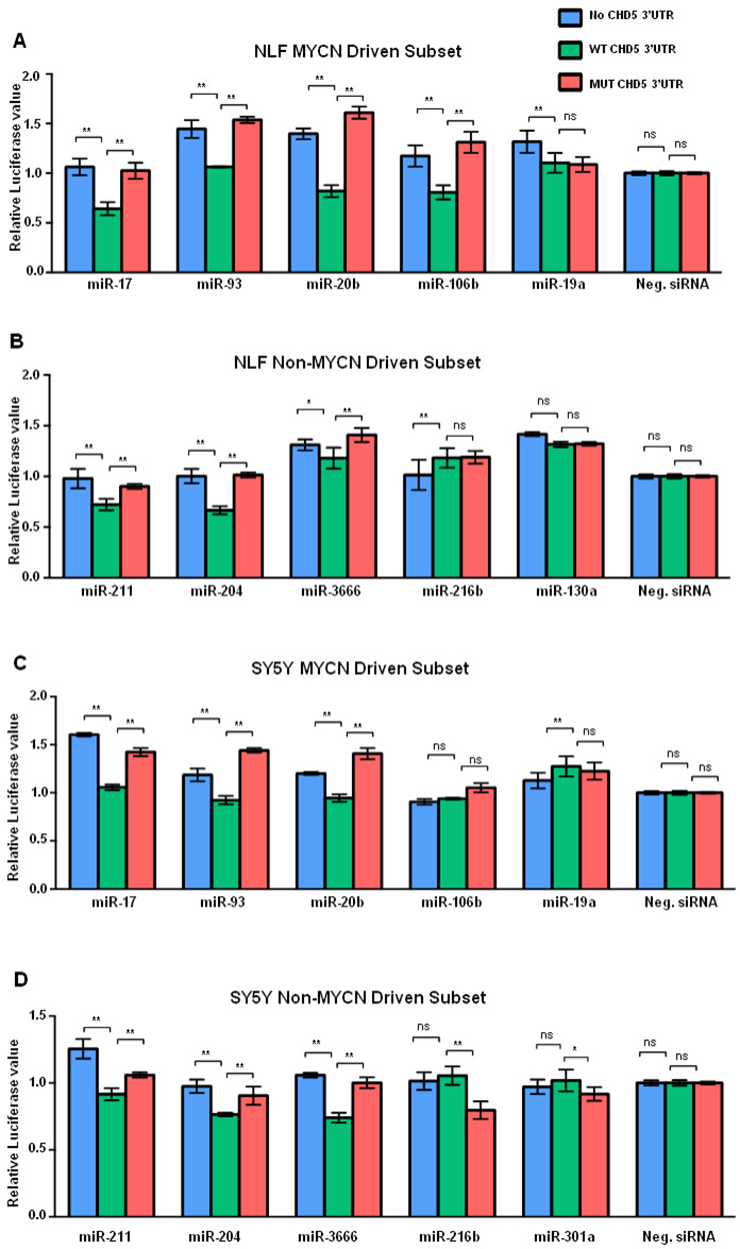
Graphic representation of miRNA regulation of *CHD5* 3′-RNA reporter construct Graphical representation of *“CHD5 levels”* in the NLF cell line after transfection with the *MYCN*-driven subset of miRNAs. Each sample was transfected with miRNAs from the *MYCN* driven subset, and either no 3′-UTR inserted (no Insert), wild-type (WT) 3′-UTR insert, or mutated 3′-UTR insert (MUT). Therefore, each value represented in the bar graph reflects the ratio of *Renilla* to firefly normalized to Allstars siRNA. **A.** NLF and *MYCN*-driven miRNAs. **B.** NLF and non-*MYCN*-driven miRNAs. **C.** SY5Y and *MYCN*-driven miRNAs. **D.** SY5Y and non-*MYCN*-driven miRNAs. Each experiment included miR-211 mimic as a positive control, Allstars siRNA (Qiagen) as a negative control, and no miRNA mimic as a transfection control. Each transfection was carried out in triplicate, and each experiment was repeated at least 3 times. Statistical analyses were performed using the Prism two-way ANOVA method followed by a Sidak post-test. Data are expressed as the standard error mean (SEM). Values are the mean of triplicates readings from four independent experiments and p-values were reported (* P<0.05, ** p<0.01 and ns= non-significant).

**Figure 3:**
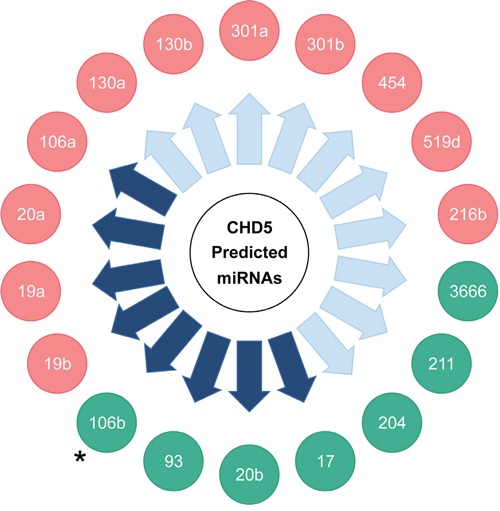
Summary of miRNA regulation of a *CHD5* 3′-UTR reporter construct in NLF and SY5Y lines with miRNA mimics predicted to target *CHD5* miRNAs in green circles caused downregulation of the *CHD5* 3′-UTR target construct in at least one of the two lines. miRNAs in red circles did not significantly downregulate the *CHD5* 3′-UTR. Purple arrows indicate miRNAs shown to be upregulated by MYC/*MYCN* expression. Asterisk (•): miRNA 106b downregulated *CHD5* 3′-UTR in NLF only, not in SY5Y.

We identified seven miRNAs (of the 18 tested) that regulated the *CHD5* 3′-UTR reporter in at least one cell line, and six were common to both the NLF and SY5Y cell lines (miR-93, -20b, -17, -204, -211, and -3666); this included the miR-211 positive control, which was predicted to bind to the *CHD5* 3′-UTR. In the NLF line, miR-106b also regulated expression of the *CHD5* 3′-UTR reporter (Figure 2A, 2B). Of the *MYCN* driven subset, miR-17, -20b, -93, and -106b all showed a significant downregulation of WT *CHD5* 3′-UTR, as measured by the luciferase assay (Figure 2B). In the SY5Y cell line, the six common miRNAs mentioned above also significantly downregulated the *CHD5* 3′-UTR reporter, but miR106b results did not reach statistical significance (Figure 2C, 2D). Nevertheless, the *MYCN*-driven miRNA -17, -20b and 93 all regulated the WT *CHD5* 3′-UTR reporter in SY5Y (Figure 2D). Thus, six of the 18 miRNAs predicted to bind the WT *CHD5* 3′-UTR downregulated expression of the reporter in both cell lines, three of the eight *MYCN*-driven miRNAs, and a fourth *MYCN*-driven miRNA (miR-106b) also regulated the reporter in NLF (Figure 3). Relative luciferase expression of all individual microRNAs in both NLF and SY5Y cell lines are shown in Supplementary Figures S1 and S2.

### Effect of miRNAs on *CHD5* protein expression

**Figure 4:**
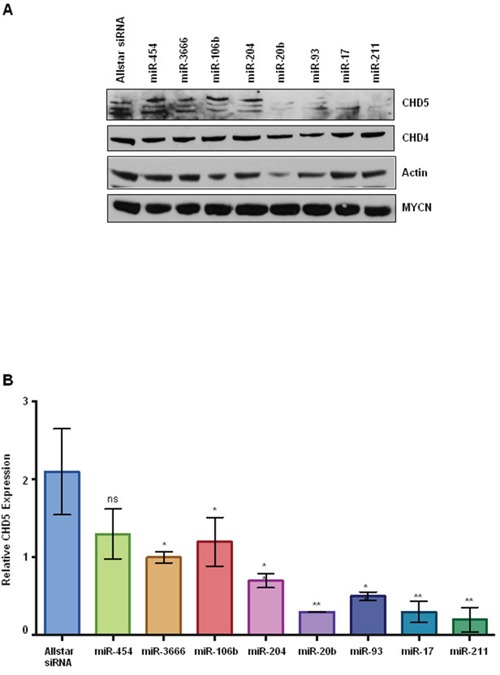
*CHD5* protein expression in NBLS cells following transient transfection with miRNA mimics **A.** Western blot analysis **of** transfected cells with indicated microRNAs. Post transfection, cells were washed twice with PBS and isolated cell extracts as described in methods [41]. Whole cell extracts (100 μg) either transfected with indicated miRNAs or mock transfected were subjected to polyacrylamide gel electrophoresis (4-12% SDS-PAGE), using NuPAGE Bis-Tris gels with MOPS-SDS Running Buffer Allstars siRNA and miRNA-454 were used as negative controls. Proteins were transferred on to nitrocellulose membranes (GE Healthcare Life Sciences) and probed with antibodies using rabbit polyclonal *CHD5*, actin (Santa Cruz Biotechnology, CA 1:1000), rabbit polyclonal CHD4 (Bethyl 1:2000), and *MYCN* monoclonal (1:5000; BD Biosciences). Almost complete reduction of *CHD5* protein levels were observed for miR-20b, miR-93, miR-17, and miR-211 as indicated, but no change in CHD4, actin or *MYCN* levels were seen. **B.** Densitometric analysis of *CHD5* protein expression in NBLS cell line. The number of pixels from each band was measured, and a bar graph was created using the Prism to indicate the difference in *CHD5* expression upon miRNA transfection. Data are expressed as the standard error mean (SEM). Statistical analysis was performed using the Prism one way ANOVA method followed by Tukey's post-test. Statistical significance relative to the control Allstar siRNA is indicated: *p<0.05; **p<0.01.

In order to further validate the functional regulation of *CHD5* expression by miRNAs, we performed transient transfections in the NBLS NB cell line, which has the highest levels of endogenous *CHD5* of the lines tested. Cells were transfected with miRNA mimics for each of the seven miRNAs identified as regulating *CHD5* expression in our reporter assay, as well as miR-454 as a negative control. Whole cell extracts were subjected to SDS-PAGE followed by western blot analysis. Our western results indicate there was almost complete reduction of *CHD5* protein levels in NBLS cells for miR-211, miR-17, miR-93 and miR-20b, whereas no change in CHD4, actin or *MYCN* protein levels were observed (Figure 4). These results strongly suggest that miR-211, -17, -93 and -20b can dramatically regulate *CHD5* protein expression in NBs.

### Analysis of miRNA, *MYCN* and *CHD5* levels of primary NB tumors

In order to understand possible *in vivo* relationships between miRNA, *MYCN* and *CHD5* expression, we analyzed 160 primary NB tumor samples. Subcellular protein extraction and western analysis were performed as described previously [37, 38]. Expression level data were available for the following miRNAs, miR-130ab, -93, -17, -106ab, -301ab, -216b, -3666, -211, -204, -20b, and -519d. We found a significant inverse relationship between *MYCN* expression and *CHD5* expression, as reported previously (data not shown) [17, 25]. We found a positive correlation between *MYCN* and three miRNAs, miR-93, -106b and -130b, two of which are considered *MYCN*-driven miRNAs. Next, we compared the *CHD5* expression levels in all 160 samples to the expression level of each miRNA. The most significant correlation we were able to find was of that between *CHD5* and miRNA 130a (data not shown). The lack of a clear correlation between the miRNAs identified here and the primary tumor data may reflect the complexities of epigenetic regulation of *CHD5* expression in primary NBs.

### miRNA regulation of *CHD5* expression in neuroblastoma and other cancers

Cai and colleagues published a study showing that miR-211 regulated *CHD5* expression *in vitro* and *in vivo* in colorectal cancer [36]. The prediction algorithms we used also identified miR-211 as targeting the *CHD5* 3′-UTR, and our reporter assay confirmed function regulation in both NB cell lines tested. Yu and coworkers recently identified miR-454 as a regulator of *CHD5* expression in hepatocellular carcinoma [39]. However, our studies did not identify activity of miR-454 in NBs, even though this miRNA was also predicted to bind to the *CHD5* 3′-UTR. Nevertheless, our reporter assay was performed using transient transfection assays, compared to permanent transfection used by Yu for their study. Also, there may be differences in the miRNAs that regulate *CHD5* in different cell types (hepatocellular carcinoma vs. NB). Nevertheless, we validated the effect of miR-211 and identified six additional miRNAs that regulate *CHD5* expression, including four *MYCN*-driven miRNAs. Interestingly, there is an inverse relationship between *MYCN* amplification/overexpression and *CHD5* expression in neuroblastomas [11, 17, 25, 40]]. Therefore, increased expression of *MYCN* could drive expression of miRNAs, which in turn could reduce the expression of *CHD5* mRNA and protein.

We used four different prediction algorithms to identify miRNAs that were likely to bind to the *CHD5* 3′-UTR, and we focused on the 18 miRNAs that were identified by at least two programs. Indeed, we provide functional evidence that at least seven of these miRNAs (including miRNA-211) regulate *CHD5* expression in at least one NB line in our reporter assay. Furthermore, four of the seven miRNAs also dramatically reduced *CHD5* protein expression in an NB line with exogenous *CHD5* expression. However, prediction algorithms are imperfect, and we may have missed other important miRNA regulators of *CHD5* expression.

Clearly there are a number of potential mechanisms of epigenetic regulation of genes, including DNA methylation, histone modification, and microRNA regulation. These may be particularly important for silencing of the remaining allele of TSGs in cancers in which one copy is deleted, but the remaining allele is not structurally inactivated. Our data strongly support miRNA regulation as an additional mechanism of *CHD5* regulation. Therapy targeting these miRNAs thus represents a potentially viable approach to reactivate expression of the remaining unmutated *CHD5* gene and restore growth control in NBs.

## MATERIALS AND METHODS

### Reagents

Cell culture media RPMI-1640 (Roswell Park Memorial Institute medium), antibiotics and fetal bovine serum (FBS) were obtained from Invitrogen Inc. (Grand Island, NY). Parental NB cell lines were maintained in our lab but are also available from the American Type Culture Collection (ATCC, Manassas, VA). 1X Dulbecco's Phosphate Buffered Saline (PBS) and Opti-MEM were purchased from Life Technologies (Grand Island, NY). Restriction enzymes and other molecular biology reagents were purchased from Roche Applied Sciences (Indianapolis, IN), Promega Inc. (Madison, WI), and New England Biolabs Inc. (Beverly, MA). NuPAGE gels (4-12%), buffers, and prestained Rainbow molecular weight markers were obtained from Invitrogen (Grand Island, NY). The Dual-Luciferase Reporter Assay reagent (E1910) was from Promega Inc. (Madison, WI).

### Antibodies

Rabbit polyclonal *CHD5* (sc-68390), rabbit polyclonal CHD4 (Bethyl 1:2000), and mouse monoclonal *MYCN* (1:5000; BD Biosciences) antibodies were used in this study. HRP conjugated secondary antibodies (1:3000) were from (GE Healthcare Life Sciences, Piscataway, NJ).

### Cell culture

We cultured NLF and SY5Y NB lines as described previously [17, 41]. NLF, SH-SY5Y neuroblastoma parental cell lines were obtained from ATCC (Manassas, VA) and cultured in RPMI-1640 with 10% FBS, 1% L-glutamine and Pen Strep (Life Technologies, Grand Island, NY) according to the provider instructions. Both cell lines were maintained at 37°C in 5% CO2 and 95% humidity. Cell authenticity was checked on annual basis by utilizing PCR techniques for mycoplasmas, bacterial and other viral contaminations as well as for genetic variations. These tests were performed at the cell center facility of the University of Pennsylvania.

### Transfections

We transfected NLF (*MYCN* amplified) and SH-SY5Y (non-amplified) neuroblastoma cell lines using commercially available lipid based reagent Lipofectamine 3000 as per the manufacturers recommendations (Invitrogen, Grand Island, NY). Briefly, cells (1×10^5^ per/well) were plated in triplicate in 24-well tissue culture plates. The next day, cells were transfected with the following: Psicheck™-2 plasmid, either wild type *CHD5* 3′-UTR, or mutated *CHD5* 3′-UTR miRNA along with various miRNA mimics (Sigma-Aldrich, St. Louis, MO) separately. PcDNA3.1 (Invitrogen, Grand Island, NY) was added as filler to match the uniform DNA concentration in each transfection mix. Transfections in triplicate were performed using Opti-MEM medium and were optimized to each cell line. Each transfection included, miR-211 mimic as a positive control, Allstars siRNA (Qiagen, Valencia, CA) as a negative control, and a no miRNA mimic condition as a transfection control. After at least 36 hours of transfection cells were washed twice with PBS, harvested cells with commercially available reagents (ThermoFisher Scientific, Waltham, MA) [41] and extracts were analyzed using the Dual-Luciferase Reporter Assay (Promega, Madison, WI) as per the manufacturer's recommendation. Each transfection was carried out in triplicate and each experiment was performed at least 3 times.

### Whole cell extract preparation

Whole cell extracts for luciferase assays and western blot analysis were prepared using commercially available kits (Promega Inc. Madison, WI and ThermoFisher Scientific, Waltham, MA) as per the manufacturer's instructions and also as described previously in Kolla et al. [41]. Protein concentrations were determined using Bradford Protein Assay Reagent with SmartSpec Plus spectrophotometer (BIO-RAD Laboratories, Hercules, CA) as described earlier by Laemmli [37].

### Luciferase reporter assay

A luciferase reporter assays were performed to verify the binding of various microRNAs that bind 3′-UTR of *CHD5* mRNA. A portion of the 3′ untranslated region of *CHD5* was cloned in a PsiCHECK, a dual reporter (Firefly and Renilla) luciferase vector (Promega Inc. Madison, WI). Transfections were performed as described above. Triplicate values of dual luciferase expressions were measured post 48 hours of transfection using a chemiluminescence plate reader (BioTek, Synergy2 multi-mode plate reader). Firefly luciferase expression values were normalized with reporter Renilla luciferase numbers to normalize the transfection efficiency in each well.

### Western analysis

Whole cell extracts (100 μg), either transfected with indicated miRNAs or mock transfected, were subjected to polyacrylamide gel electrophoresis (4-12% SDS-PAGE), using NuPAGE Bis-Tris gels with MOPS-SDS Running Buffer (Invitrogen, Grand Island, NY). Allstars siRNA and miRNA-454 were used as negative controls. Proteins were transferred on to nitrocellulose membranes (GE Healthcare Life Sciences) and probed with antibodies using rabbit polyclonal *CHD5*, actin (Santa Cruz Biotechnology, CA 1:1000), CHD4 (Bethyl laboratories, Montgomery, TX 1:2000), and *MYCN* monoclonal (1:5000; BD Biosciences, San Jose, CA). HRP conjugated secondary antibodies (1:3000) were from (GE Healthcare Life Sciences, Piscataway, NJ).

### Statistical analysis

Statistical analyses were performed using the Prism two-way ANOVA method followed by a Sidak post-test. Each experiment was performed at least three times and triplicate readings were used and reported all p-values. Data are expressed as the standard error mean (SEM). Values are the mean of triplicates readings from four independent experiments and p-values were reported (* P<0.05, ** p<0.01 and ns= non-significant).

## SUPPLEMENTARY FIGURES AND TABLES





## Abbreviations

TSG: Tumor Suppressor Gene
CHD5: Chromodomain Helicase DNA binding protein 5
NB: Neuroblastoma
SRD: Smallest Region of consistent Deletion
