# Supplementary material for: Role of microRNAs in epigenetic silencing of the CHD5 tumor suppressor gene in neuroblastomas
Source: Oncotarget. 2016 Feb 16;7(13):15977–85. doi: 10.18632/oncotarget.7434 (PMC4941291; doi:10.18632/oncotarget.7434)
Supplement: Supplementary file 1 [file oncotarget-07-15977-s001.pdf]

SUPPLEMENTARY FIGURES AND TABLE

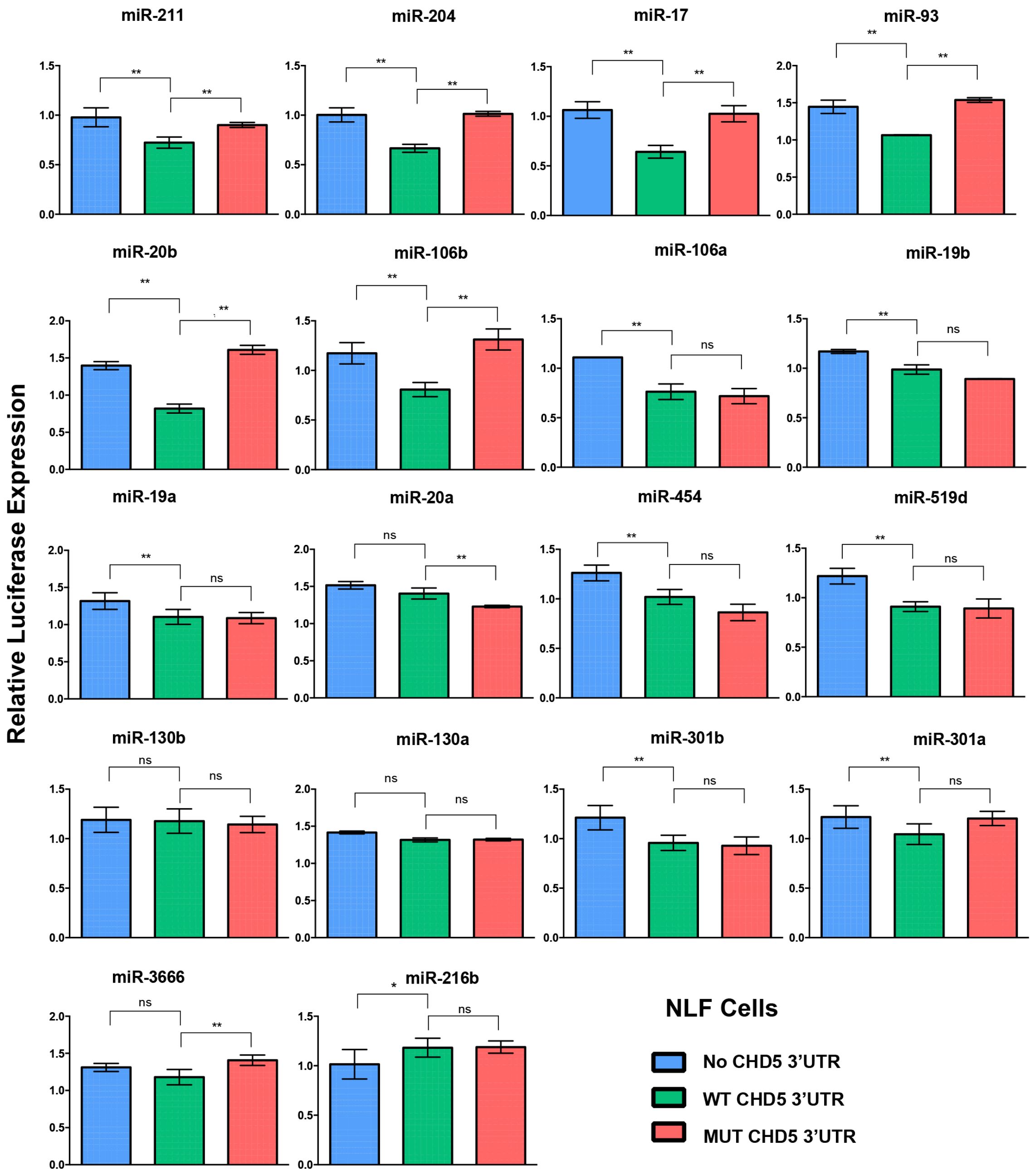

Supplementary Figure S1: Graphic representation of individual miRNAs on regulation of *CHD5* 3'RNA reporter construct in NLF cell line. Each sample was transfected with miRNAs from both the *MYCN* driven and no-*MYCN* driven subset with either no 3'-UTR inserted (No Insert), wild-type (WT) 3'-UTR insert, or mutated 3'-UTR insert (MUT). MiRNA mimic miR-211 and Allstars siRNA served as positive and negative controls respectively. Therefore, each value represented in the bar graph reflects the ratio of *Renilla* to firefly normalized to Allstars siRNA. 'Y' axis indicates relative luciferase expression. Statistical analyses were performed using the Prism two-way ANOVA method followed by a Sidak post-test. Data are expressed as the standard error mean (SEM). Values are the mean of triplicates readings from four independent experiments and p-values were reported (* P<0.05, ** p<0.01 and ns=non significant).

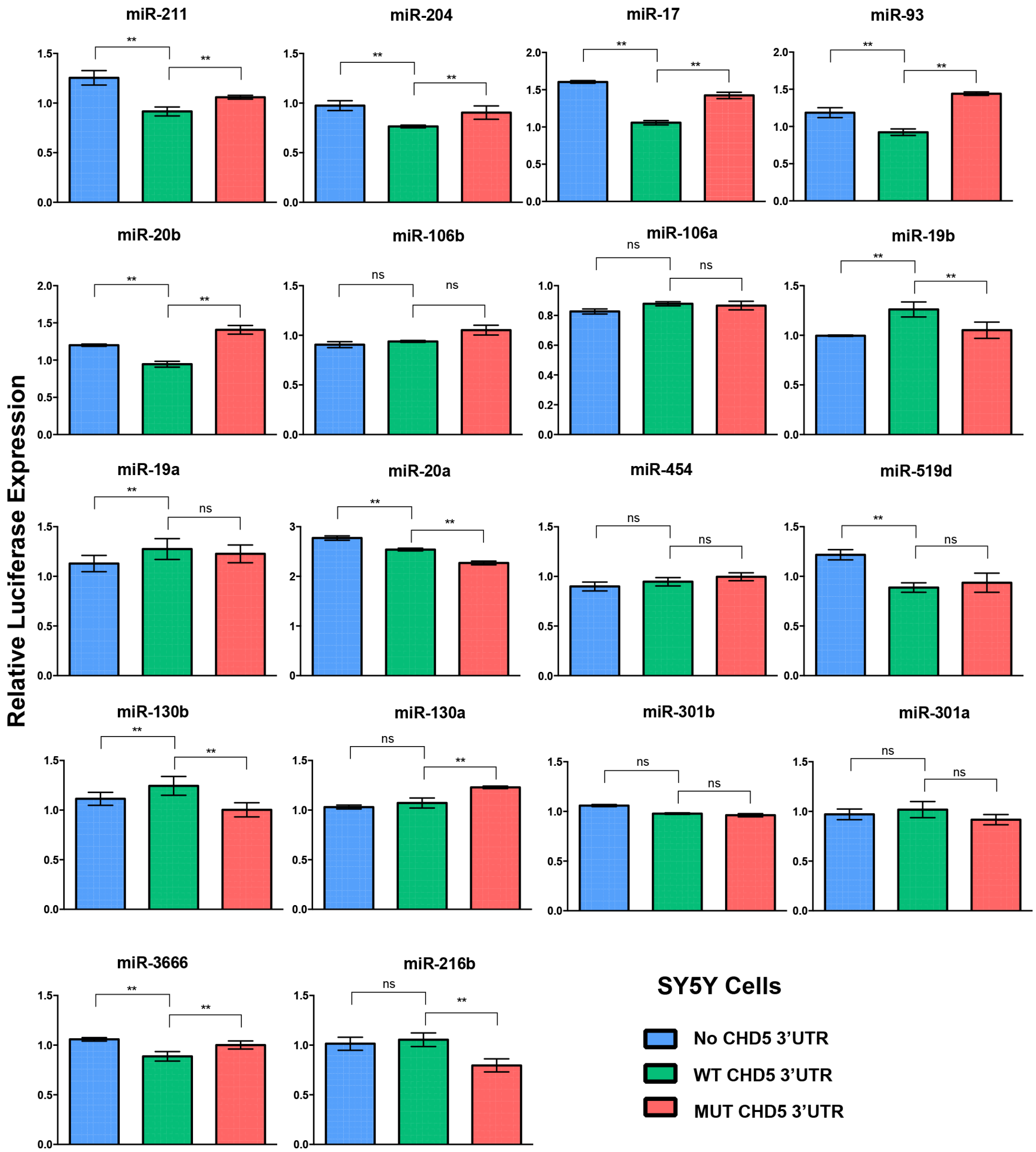

Supplementary Figure S2: Graphic representation of individual miRNAs on regulation of *CHD5* 3'RNA reporter construct in SY5Y cell line. Each sample was transfected with miRNAs from both the *MYCN* driven and no-*MYCN* driven subset with either no 3'-UTR inserted (No Insert), wild-type (WT) 3'-UTR insert, or mutated 3'-UTR insert (MUT). MiRNA mimic miR-211 and Allstars siRNA served as positive and negative controls respectively. Therefore, each value represented in the bar graph reflects the ratio of *Renilla* to firefly normalized to Allstars siRNA. 'Y' axis indicates relative luciferase expression. Statistical analyses were performed using the Prism two-way ANOVA method followed by a Sidak post-test. Data are expressed as the standard error mean (SEM). Values are the mean of triplicates readings from four independent experiments and p-values were reported (* P<0.05, ** p<0.01and s=non significant).

Supplementary Table S1: List of identified microRNAs along with their chromosomal location and nucleotide sequence

See Supplementary File S1
