# Supplementary material for: Role of microRNAs in epigenetic silencing of the CHD5 tumor suppressor gene in neuroblastomas
Source: Oncotarget. 2016 Feb 16;7(13):15977–85. doi: 10.18632/oncotarget.7434 (PMC4941291; doi:10.18632/oncotarget.7434)
Supplement: Supplementary file 2 [file oncotarget-07-15977-s002.pdf]

Supplementary Table S1. List of identified microRNAs along with their chromosomal location and nucleotide sequence.

**microRNA***	**Chromosome**	**Nucleotide sequence 5'- 3'**
hsamiR-204	9q21.12	UUCCCUUUGUCAUCCUAUGCCU
hsa-miR-211	15q13.3	UUCCCUUUGUCAUCCUUCGCCU
hsa-miR-216b	2p16.1	AAAUCUCUGCAGGCAAAUGUGA
hsa-miR-3666	7q31.1	CAGUGCAAGUGUAGAUGCCGA
hsa-miR-17	13q31.3	CAAAGUGCUUACAGUGCAGGUAG
hsa-miR-19a	13q.31.3	AGUUUUGCAUAGUUGCACUACA
hsa-miR-19b	13q.31.3	UGUGCAAAUCCAUGCAAAACUGA
hsa-miR-20a	13q.31.3	UAAAGUGCUUAUAGUGCAGGUAG
hsa-miR-20b	Xq26.2	CAAAGUGCUCAUAGUGCAGGUAG
hsa-miR-93	7q.22.1	CAAAGUGCUGUUCGUGCAGGUAG
hsa-miR-106a	Xq26.2	AAAAGUGCUUACAGUGCAGGUAG
hsa-miR-106b	7q22.1	UAAAGUGCUGACAGUGCAGAU
hsa-miR-130a	11q.12.1	UUCACAUUGUGCUACUGUCUGC
hsa-miR-130b	22q11.21	ACUCUUUCCCUGUUGCACUAC
hsa-miR-301a	17q.22	GCUCUGACUUUAUUGCACUACU
hsa-miR-301b	22q11.21	CAGUGCAAUGAUAUUGUCAAAGC
hsa-miR-454	17q.22	ACCCUAUCAAUAUUGUCUCUGC
hsa-miR-519d	1pq.13.42	CAAAGUGCCUCCCUUUAGAGUG

* Computational analysis using bioinformatic tools such as miRanda (www.microrna.org), TargetScan (www.targetscan.org), miRDB (mirdb.org), and DIANA 3.0 (diana.cslab.ece.ntua.gr/microT) were used to predict miRNAs that target 3' UTR of CHD5.
